# The value of proportion of perfused vessels change rate in the evaluation of early organ function deterioration in septic shock and ARDS

**DOI:** 10.3389/fmed.2026.1733855

**Published:** 2026-01-22

**Authors:** Shuya Huang, Xiaoyan Li, Rui Dong, Yanqiu Gao

**Affiliations:** Respiratory Intensive Care Unit (RICU), Zhengzhou Central Hospital Affiliated to Zhengzhou University, Zhengzhou, China

**Keywords:** acute respiratory distress syndrome, deterioration of organ function, lactic acid clearance, microcirculation, septic shock

## Abstract

**Background:**

Severe organ function deterioration is associated with poor prognosis in patients with septic shock combined with ARDS. This study aimed to develop a validated predictive model for early organ function deterioration and to evaluate the factors associated with this deterioration, as well as the prognosis, in patients with septic shock combined with ARDS.

**Methods:**

This is a retrospective study including 67 patients with septic shock combined with ARDS. Patients were categorized into two groups based on the change in their Sequential Organ Failure Assessment (SOFA) score over 24 h: the organ function deterioration group (SOFA_24h − 0h_ score ≥ 1) and the non-deterioration group (SOFA_24h − 0h_ score <1). The sublingual microvasculature of patients was assessed using microcirculatory microimaging to obtain metrics such as proportion of perfusion vessel change rate (ΔPPV), which were then analyzed to characterize the patients with early organ function deterioration.

**Results:**

There were a total of 34 patients with early organ function deterioration. ΔPPV and LCR were independently associated with early organ function deterioration, and ΔPPV and lactate clearance rate (LCR) were associated with ΔSOFA. The AUC for ΔPPV was 0.813 (95% CI: 0.707–0.919), and when combined with the LCR, the AUC was 0.871 (95% CI: 0.785–0.957).

**Conclusions:**

Deterioration of organ function is common in patients with septic shock combined with ARDS and early detection is crucial. Microcirculation is an important factor in safeguarding organ function. We developed a predictive model to predict the risk of early organ function deterioration, and the combination of ΔPPV and LCR may merit further investigation.

## 1 Introduction

Sepsis is caused by a dysregulated host response to infection, resulting in life-threatening organ dysfunction. Characterized by an imbalance in oxygen supply and demand, septic shock is a type of distributive shock that concurrently affects both macrocirculation and microcirculation (1, 2). The lungs, often the initial target in the cascade of multiple organ dysfunction syndrome (MODS) triggered by sepsis, are particularly susceptible to acute respiratory distress syndrome (ARDS) (3), making septic shock and ARDS significant contributors to mortality in critically ill patients.

Additionally, the severity of microcirculatory disorders are associated with increased risk of organ failure and poor prognosis (4). Interestingly, few studies have examined the direct relationship between microcirculatory disturbances and early organ functional impairment.

Trzeciak et al. found that improvements in sublingual microcirculation were associated with enhanced organ function during the early stages of septic shock resuscitation (5). Prompt improvement in microcirculation within the first 6 h can significantly reduce the likelihood of early organ failure and mortality (6). Pan et al. (7) adopted 6 h as the critical time window for evaluating microcirculatory changes and metabolic indicators in their study. Therefore, 6 h represents a pivotal and extensively researched time point for assessing early recovery responses and predicting subsequent disease progression. De Bacher et al. (8) demonstrated that the extent of microcirculatory dysfunction independently predicted survival, with the proportion of perfused vessels (PPV) being the most significant predictor.

Recent studies emphasize the dynamic changes in microvasculature following treatment, which are influenced by individual differences and potential confounding factors, making a single measurement of PPV less reliable. The rate of change in PPV provides insights into changes in vascular opening and the quality of microvascular perfusion over time and is closely associated with poor prognosis in patients with septic shock (7). However, clinical studies focusing on ΔPPV in the early deterioration of organ function in septic shock combined with ARDS are scarce. Normalization of blood lactate levels is a critical goal in the resuscitation of septic shock patients, thus lactate clearance rate (LCR) is a valuable metric for evaluating the effectiveness of treatment (9). Nguyen et al. (10) demonstrated that early lactate clearance is a strong predictor of survival in patients with sepsis.

In this study, we observed the proportion of perfused vessels change rate (ΔPPV) and LCR, analyzing their predictive value for early deterioration of organ function in patients with septic shock and ARDS. Additionally, we investigated the correlation between ΔPPV, LCR, and ΔSOFA to provide clinical insights for the management of sepsis-induced organ failure.

## 2 Materials and methods

### 2.1 Patients

A retrospective study was conducted on patients diagnosed with septic shock and ARDS who were admitted to the intensive care unit (RICU) of Zhengzhou Central Hospital, affiliated with Zhengzhou University, from October 1, 2021, to May 31, 2023. Inclusion criteria included patients who met the diagnostic standards of Sepsis-3 (11) and the ARDS Berlin Criteria (12). Exclusion criteria were age under 18 years, pregnancy, presence of malignant neoplasms, use of immunosuppressive drugs, oral diseases or heavy bleeding that could affect sample quality, inability to perform invasive mechanical ventilation, and a hospital stay of less than 24 h or death shortly after unsuccessful resuscitation. The study was conducted in accordance with the guidelines of the Declaration of Helsinki and was approved by the Ethics Committee of Zhengzhou Central Hospital affiliated with Zhengzhou University (approval number: 202408).

### 2.2 Clinical date collection

Data collected included the proportion of perfused vessels (PPV), lactate (Lac), oxygen to inspired oxygen fraction (PaO_2_/FiO_2_), central venous pressure (CVP), and mean arterial pressure (MAP) at the time of admission to the RICU (0 h) and at 6 h. Serum creatinine (SCr), total bilirubin (Tbil), C-reactive protein (CRP), procalcitonin (PCT), and platelet count (PLT) were collected on admission.

The change in perfused vessel proportion (ΔPPV) was calculated as ΔPPV = [(PPV_T_ – PPV_T0_)/PPV_T0_] × 100%; lactate clearance rate (LCR) was calculated as LCR = [(Lac_T0_ – Lac_T_)/Lac_T0_] × 100%. Acute Physiology and Chronic Health Evaluation II (APACHE II) and Sequential Organ Failure Assessment (SOFA) scores were recorded at admission and 24 h later. Patients were categorized based on the change in SOFA score over 24 h (SOFA_24h − 0h_ score), with groups defined as having organ function deterioration (SOFA_24h − 0h_ score ≥ 1) or non-deterioration (SOFA_24h − 0h_ score <1).

#### 2.2.1 Methods for measuring sublingual microcirculation

Sublingual microcirculation was assessed using the MicroSeeV100 handheld video microscope (Medsoft, Guangzhou, China). This device operates based on the principle of side-stream dark-field imaging. Image acquisition strictly followed the standardized operating procedures outlined in the 2018 European Society of Intensive Care Medicine (ESICM) Microcirculation Consensus Statement (13):

Operator training and patient preparation: prior to data collection, the operator completed a structured training program that included theoretical instruction on microcirculatory physiology, hands-on practice on healthy volunteers, and calibration against reference videos to ensure consistent probe handling and artifact recognition. Sublingual secretions were gently removed using a cotton swab moistened with 37 °C saline. The probe, fitted with a disposable sterile cap, was placed lightly on the sublingual mucosa bilaterally to the lingual frenulum, with careful avoidance of any pressure to prevent compression-induced flow artifacts.

Image acquisition time window: to assess microcirculatory responses during early recovery, measurements were taken at patient admission to the RICU (0 h) and 6 h after initiating standard therapy. Each measurement occurred at least 15 min after stabilization of hemodynamic interventions (e.g., rapid fluid resuscitation, vasoactive drug adjustments) to ensure images reflected a relatively stable physiological state.

Acquisition protocol: video sequences were captured from at least five distinct fields of view on both sides of the lingual frenulum. Each sequence lasted a minimum of 20 s to ensure intermittent blood flow capture and sufficient frames for spatiotemporal analysis.

Real-time image quality assessment: operators confirm during acquisition that individual red blood cell movement is clearly observable within capillaries, ensuring uniform illumination, sharp focus, and absence of saliva or bubble obstruction in the field of view.

Image quality control (MIQS): the obtained video clips were sent to two trained physicians, who immediately scored them using the MIQS developed by Massey et al. (14). MIQS comprises lighting, duration, focus, content, stability, and pressure. The two physicians were unaware of the patient's clinical status. If either physician deemed a video clip unacceptable, a new clip was immediately captured and submitted to both physicians for re-evaluation. This process continued until all five video clips received acceptable or good quality scores, at which point the unacceptable clips were discarded.

Data analysis: the screened videos were analyzed by two physicians blinded to the clinical data of the patients, using a sublingual microcirculation imaging system (Model V100, Version V1; Medsoft, Guangzhou, China). The captured video is divided into four independent quadrants, each evaluated based on microvascular blood flow characteristics. A standardized scoring system is applied: no blood flow (score 0), intermittent blood flow (score 1), slow blood flow (score 2), normal blood flow (score 3). The mean score across the four quadrants yields a quantitative indicator of overall microcirculatory perfusion: the Microcirculatory Flow Index (MFI). Perfusion Pressure Ratio (PPV) is calculated as the ratio of perfused microvascular length to total microvascular length, serving as an indicator of microcirculatory perfusion quality.

#### 2.2.2 Measurement of hemodynamic indicators

Intracervical or subclavian venous catheters (7FR, Weihai Jiwei Intensive Care Medical Products Co., Ltd., China) were placed in all patients, and hemodynamic measurements were performed using IntelliVue MP60 monitors (Philips, Germany). Monitored parameters included heart rate (HR), central venous pressure (CVP), and mean arterial pressure (MAP).

### 2.3 Statistical analysis

Data were analyzed using SPSS 26.0 (IBM, Armonk, NY, USA) and GraphPad Prism 9.4.1.681 (GraphPad Software, Boston, USA). Continuous variables following a normal distribution were expressed as mean ± standard deviation, and comparisons between groups were made using the *t*-test. Variables not normally distributed were described using the median and interquartile range (IQR), with the Mann-Whitney *u*-test applied for intergroup comparisons. Categorical variables were summarized as counts and percentages, and analyzed using the Chi-square or Fisher's exact test as appropriate. Correlations were assessed using the Spearman rho statistic. The receiver operating characteristic (ROC) curve analysis was used to evaluate the discriminative ability of the clinical predictors. The 28-day mortality rates were calculated, and survival curves were plotted using the Kaplan-Meier method, with the Log-rank test assessing differences among predefined groups. A *P*-value <0.05 was considered statistically significant.

## 3 Results

### 3.1 General characteristics

We initially collected 106 patients with a confirmed diagnosis of septic shock and ARDS. After screening, seven patients were excluded due to death or discharge within 24 h of admission to the intensive care unit. Additionally, nine patients with malignant tumors, six patients receiving immunosuppressive therapy, four patients with oral diseases or hemorrhage, and 13 patients unable to undergo invasive mechanical ventilation were also excluded. Ultimately, 67 patients were included in the study −43 males and 24 females—with an average age of 74.12 ± 14.01 years. Based on the 24-h change in SOFA scores, patients were classified into two groups: the deterioration group (*n* = 34) and the non-deterioration group (*n* = 33). The overall ΔSOFA (24-h minus 0-h) across all patients had a median of 0.00 (IQR: −1.00 to 2.00). In the deterioration group, the median ΔSOFA was 2.00 (IQR: 1.00 to 2.25), while in the non-deterioration group, it was −1.00 (IQR: −2.00 to 0.00). Tbil and PCT in the deteriorating group were significantly different from those in the non-deteriorating group. Otherwise, no significant statistical differences were observed in the baseline characteristics between these groups (*P* > 0.05; see Table 1).

**Table 1:** Characteristics of the study population.

**Characteristics**	**Deterioration (*n* = 34)**	**Non-deterioration (*n* = 33)**	***P***
Age (years)	71.94 ± 15.58	76.36 ± 12.02	0.199
Males	22 (64.7)	21 (63.6)	0.927
Temperature (°C)	37.40 ± 1.06	37.60 ± 0.99	0.435
Respiratory rate (breaths/min)	21.86 ± 4.59	21.00 ± 3.35	0.387
**Comorbidities**, ***n*** **(%)**			
Hypertension	11 (32.4)	15 (45.5)	0.271
Diabetes	10 (29.4)	9 (27.3)	0.846
Cardiovascular	16 (47.1)	15 (45.5)	0.895
Respiratory	14 (41.2)	10 (30.3)	0.353
**Site of infection**, ***n*** **(%)**			0.914
Respiratory tract	11 (32.4)	14 (42.4)	
Intra-abdominal	5 (14.7)	4 (12.1)	
Urinary tract	5 (14.7)	4 (12.1)	
Skin/soft tissue	9 (26.5)	6 (18.2)	
Others	4 (11.8)	5 (15.2)	
APACHE II score	24.23 ± 5.86	22.82 ± 5.24	0.301
**SOFA score**			
0 h	12.59 ± 2.56	12.15 ± 2.77	0.505
24 h	14.53 ± 2.51	11.33 ± 2.90	<0.001^*^
ΔSOFA	2 (1.00, 2.25)	−1 (−2.00, 0.00)	<0.001^*^
SCr (μ mol/L)	127.50 ± 84.25	104.97 ± 71.24	0.242
Tbil (μ mol/L)	23.65 (17.68, 43.13)	19.60 (9.70,27.10)	0.009^*^
CRP (mg/L)	119.45 ± 82.40	94.63 ± 72.82	0.196
PCT (ng/L)	28.17 ± 42.50	8.19 ± 13.36	0.013^*^
PLT (× 10^9^/L)	113.41 ± 80.10	133.24 ± 81.55	0.319
Length of stay RICU (days)	16.47 ± 13.41	17.12 ± 11.61	0.833

Data are presented as means (standard deviation; ± SD), median (interquartile range; IQR), or counts (percentage). Differences were tested using the Kruskal–Wallis test or Fisher exact test unless indicated otherwise. APACHE-II, acute physiology and chronic health evaluation II; SCr, serum creatinine; Tbil, total bilirubin; CRP, c-reactive protein; PCT, procalcitonin; PLT, blood platelet; RICU, ICU, respiratory intensive care unit. ^*^P < 0.05.

### 3.2 Hemodynamic and microcirculatory perfusion indicators

At the outset and 6 h post-admission, there were no significant differences in heart rate (HR), central venous pressure (CVP), mean arterial pressure (MAP), and PaO_2_/FiO_2_ ratios between the two groups (*P* > 0.05). Similarly, no significant differences were noted in initial PPV values. However, after 6 h of treatment, the PPV was significantly lower in the deteriorated group compared to the non-deteriorated group, although it remained higher than the initial value (*P* < 0.05; see Table 2). Additionally, both ΔPPV and LCR were lower in the deterioration group compared to the non-deterioration group (*P* < 0.05). We classified the patients into mild, moderate, and severe ARDS categories and compared the respective metrics across these groups. A statistically significant difference in ΔPPV was observed between the severe and moderate ARDS groups (Supplementary Table E1).

**Table 2:** Comparison of patient indicators between the two groups.

**Characteristics**		**Deterioration (*n* = 34)**	**Non-deterioration (*n* = 33)**	***P***
HR (min-1)	0 h	100.59 ± 8.00	97.24 ± 7.75	0.087
	6 h	97.50 ± 12.98	93.88 ± 6.13	1.149
CVP (cmH_2_O)	0 h	9.32 ± 2.77	9.12 ± 1.43	0.710
	6 h	9.38 ± 2.28	9.27 ± 1.35	0.811
MAP (mmHg)	0 h	73.36 ± 13.97	73.44 ± 11.79	0.981
	6 h	79.61 ± 14.98	81.09 ± 10.58	0.641
PaO_2_/FiO_2_	0 h	186.32 ± 70.08	187.24 ± 62.16	0.955
	6 h	218.78 ± 89.58	231.86 ± 65.62	0.499
Lac (mmol/L)	0 h	7.3 (5.28–9.65)	5.1 (3.90–6.75)	0.026^*^
	6 h	6.25 (4.52–8.75)	4.00 (3.15–5.45)	0.001^*^
PPV %	0 h	70.61 ± 11.94	72.28 ± 12.25	0.574
	6 h	74.54 ± 11.58	81.18 ± 12.54	0.028^*^
LCR	-	13.41 (4.06–20.71)	17.65 (9.71–29.75)	0.003^*^
ΔPPV	-	6.41 (3.08–8.78)	11.66 (8.75–14.07)	<0.001^*^

HR, heart rate; CVP, central venous pressure; MAP, mean arterial pressure; PaO_2_/FiO_2_-ratio, pressure of oxygen/fraction of inspired oxygen-ratio; PPV, the proportion of perfused vessels; ΔPPV, the proportion of perfused vessels change rate; LCR, lactate clearance rate. ^*^P < 0.05.

### 3.3 Independent factors influencing early organ function deterioration in patients with septic shock and ARDS

**Figure 1:**
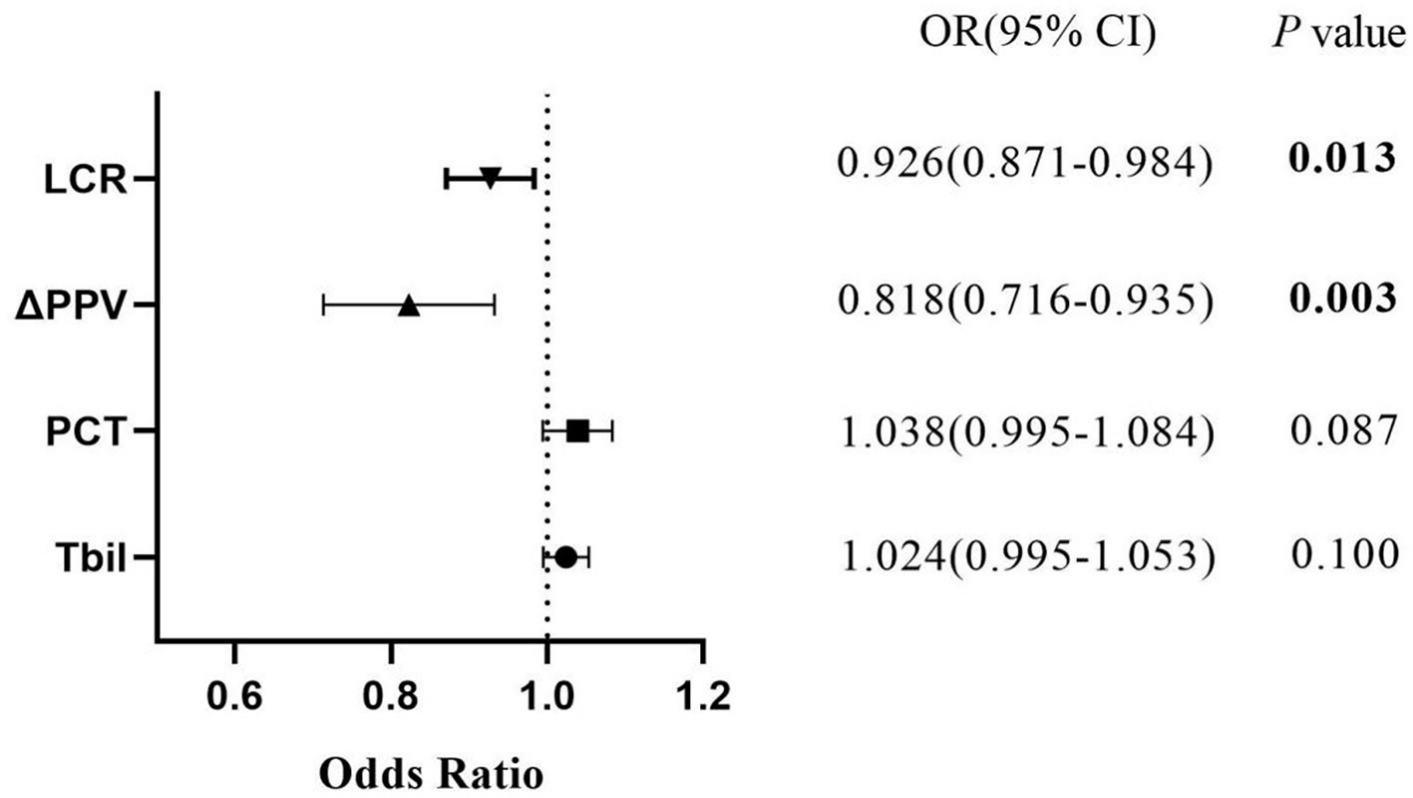
Independent factors associated with early organ function deterioration in patients.

Multivariate logistic regression analysis revealed that factors including ΔPPV and LCR were independently associated with the onset of early organ function deterioration (see Figure 1, Table 3).

**Table 3:** Results of multivariate analysis in patients.

**Variable**	**OR**	**95% CI**	***P***
LCR	0.926	0.871–0.984	0.013^*^
ΔPPV	0.818	0.716–0.935	0.003^*^
PCT	1.038	0.995–1.084	0.087
Tbil	1.024	0.995–1.053	0.100

LCR, lactate clearance rate; ΔPPV, the proportion of perfused vessels change rate; PCT, procalcitonin; Tbil, total bilirubin. ^*^P < 0.05.

### 3.4 ΔPPV and LCR are important parameters for assessing early organ function deterioration.

**Figure 2:**
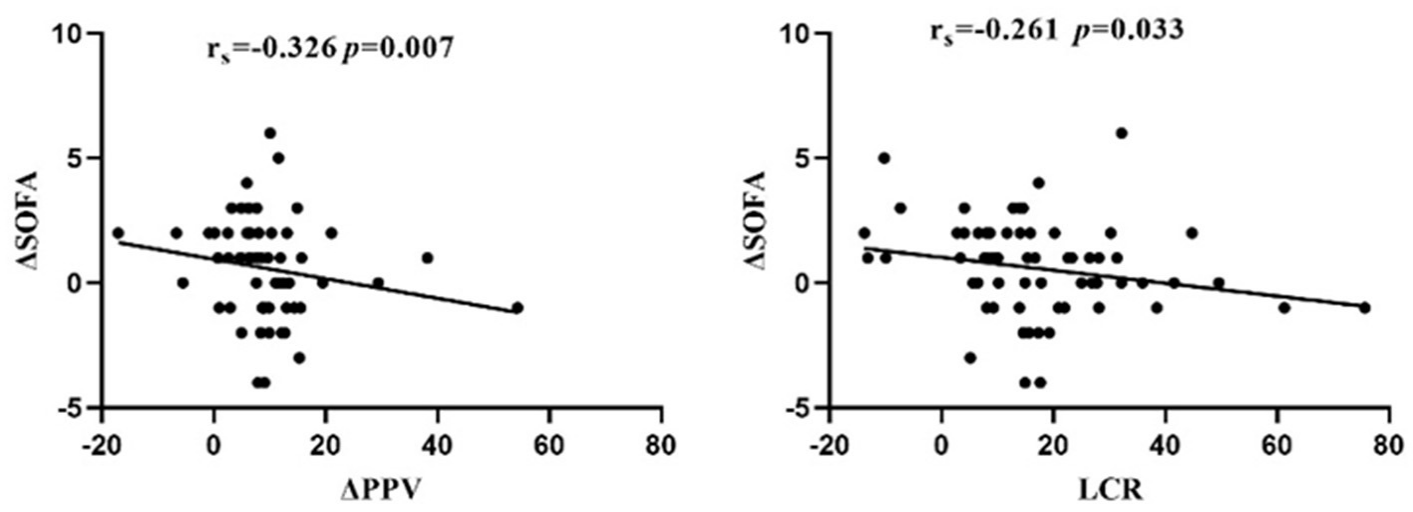
ΔPPV and LCR are important parameters for assessing early organ function deterioration.

Based on the above analysis in Table 2, we found that ΔPPV and LCR were significantly lower in the early organ function deterioration group, and both were independent correlates of early organ function deterioration in patients with septic shock combined with ARDS. We further performed a correlation analysis between ΔPPV, LCR and ΔSOFA (SOFA_24h − 0h_) and found that ΔPPV, LCR and ΔSOFA were all negatively correlated (*r*_s_ = −0.326, −0.261; *p* = 0.007, 0.033; see Figure 2).

### 3.5 Predictive test of early organ function deterioration

**Figure 3:**
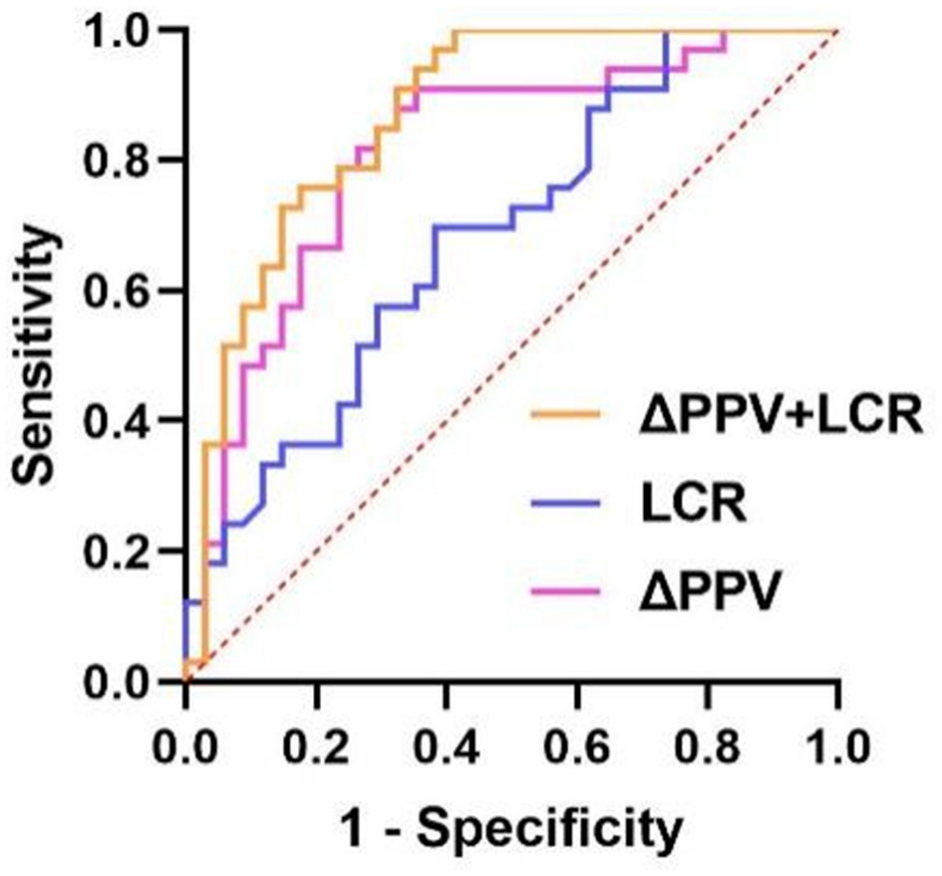
ROC curves for predicting early organ function deterioration in patients.

ROC analysis indicated that ΔPPV possesses predictive value for early organ function deterioration in patients with septic shock and ARDS. The combined area under the curve (AUC) for ΔPPV and LCR was 0.871, with a sensitivity of 61.80% and a specificity of 97.00% (see Figure 3, Table 4).

**Table 4:** Predictive value of single and combined indicators in the early organ function deterioration.

**Variable**	**AUC (95% CI)**	**Sensitivity (%)**	**Specificity (%)**	**Cutoff**	***P***
ΔPPV	0.813 (0.707, 0.919)	90.90	64.70	7.642	<0.0001^*^
LCR	0.685 (0.559, 0.811)	69.70	38.20	14.557	0.009^*^
ΔPPV+ LCR	0.871 (0.789, 0.957)	61.80	97.00	–	<0.0001^*^

ROC, receiver operating characteristic; AUC, area under the curve.^*^P < 0.05.

### 3.6 Survival curves of patients in patients with septic shock and ARDS

**Figure 4:**
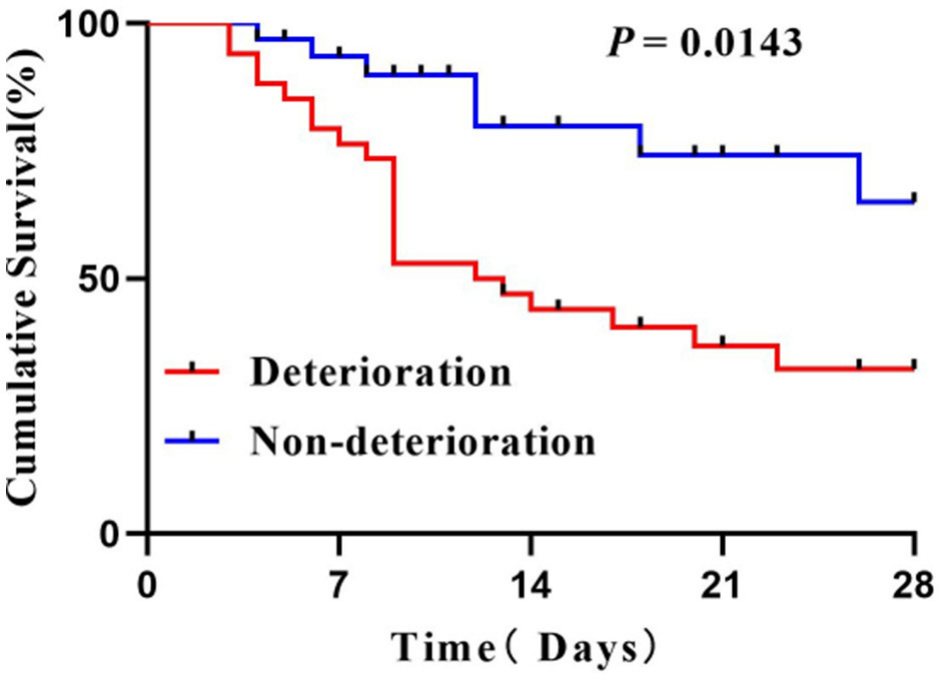
Survival curves of patients.

Cumulative survival was comparable between the two groups. The patients with early organ failure tending to have shorter 28-day cumulative survival (Log-rank test, *P* = 0.0143; see Figure 4).

## 4 Discussion

Organ failure is a critical event in the pathogenesis of sepsis, marking a hallmark of the disease and a pivotal determinant of prognosis. The damage induced by sepsis stems from the persistence of a mismatch between perfusion and tissue metabolic demands (15). Inflammatory responses leading to cardiac dysfunction and systemic volume redistribution are central to this mismatch, but the situation is exacerbated by impaired tissue oxygen utilization (16). Early indicators of organ function deterioration are vital for prognosis, as they represent the most significant changes in the disease's course. Thus, the prompt, accurate assessment of macrocirculation-microcirculation coupling, and clarification of patient conditions are essential for developing personalized blood flow management strategies to reduce organ failure rates and improve outcomes (17). Our study defines “early organ function deterioration” as ΔSOFA ≥ 1, with this threshold designed to capture early, subtle functional changes for early warning purposes. This is consistent with the definition used by Zeng et al. (18) in a similar microcirculation study. However, we acknowledge that while this threshold exhibits high sensitivity, its specificity may be insufficient. It could potentially include transient, clinically insignificant fluctuations in SOFA scores within the deterioration group, thereby affecting the specificity of the results.

Recent advances in visual imaging technology have enabled the direct observation of capillary changes at the bedside. Studies confirm that alterations in sublingual microcirculation mirror changes in other vital organs, such as the intestines and kidneys (19, 20). Therefore, microcirculation imaging systems used to analyze sublingual microcirculation parameters effectively evaluate the early and ongoing deterioration of critically ill patients and guide precise, individualized treatment strategies. Research by Sakr et al. (21) found that microcirculatory perfusion, continuously impaired in non-survivors of multiple organ failure, improved rapidly in survivors. Our study corroborates these findings, showing uncoupling of microcirculation from macrocirculation after early resuscitation, with sustained hypoperfusion despite normal macrocirculation indices. Patients whose organ function did not deteriorate exhibited significantly higher ΔPPV and PPV during early resuscitation, suggesting that disturbances in microcirculation induced by sepsis are a likely mechanism behind multiorgan dysfunction. Even when initial microcirculatory abnormalities are corrected, improved responses can gradually reduce the likelihood of organ function deterioration (22). Trzeciak et al. also demonstrated experimentally that microcirculatory disturbances occur independently of macrocirculatory changes, with early fluid resuscitation enhancing microcirculatory flow and reducing organ failure within 24 h (5). Levy et al. (23) indicates that improvement in organ function within the first 24 h after treatment initiation is significantly correlated with 28-day survival rates. This emphasizes the importance of assessing patients' dynamic response during the earliest treatment phase (within 24 h). Although shorter time windows (e.g., 6 h) may reflect hemodynamic or metabolic responses earlier, organ function cannot fully reflect the overall therapeutic response within such a short timeframe. Delaying assessment and intervention until 48 h may miss the optimal treatment window, adversely affecting prognosis. Therefore, the 24-h window achieves an optimal balance between sensitivity, specificity, and clinical feasibility, making it suitable for this exploratory study.

Abnormal microcirculatory perfusion often coincides with impaired cellular metabolic energy. Although cell metabolism indices reflect tissue cellular metabolism, they also indirectly indicate microcirculatory function. Occasionally, despite optimal resuscitation and life support, elevated lactate levels persist, possibly due to mitochondrial dysfunction triggered by sepsis (24). Effective lactate clearance requires not only sufficient macrocirculatory blood flow to provide oxygen and reduce anaerobic fermentation but also robust microcirculatory function for effective lactate metabolism. Our findings reveal that the lactate clearance rate (LCR) is higher in patients without early organ function deterioration, suggesting that mitochondrial dysfunction is another critical factor contributing to organ dysfunction during sepsis. Previous studies have shown that both human and animal models exhibit improved organ function and survival rates when mitochondrial function is enhanced (25–27). Our findings demonstrate that both ΔPPV and lactate clearance rate (LCR) are independently and negatively associated with early organ dysfunction, supporting a dual-pathway model of sepsis-induced organ failure. Specifically, ΔPPV reflects dynamic microcirculatory responsiveness—its reduction indicating impaired capillary perfusion despite adequate macrocirculation—while LCR integrates systemic perfusion adequacy with cellular metabolic capacity, particularly mitochondrial function. The negative correlation between these parameters and worsening organ function underscores their pathophysiological relevance. Both ΔPPV and LCR proved to be valuable in predicting early organ dysfunction; however, their combination enhanced predictive efficacy. Although the sensitivity has decreased, the specificity has improved, indicating that the combined index can more accurately identify patients with relatively normal microcirculation and organ function. This may provide more clinically significant predictions in practice, particularly for individuals at higher risk. Pan et al. (7) noted that 28-day mortality rates are elevated in septic shock patients with ΔPPV below 12.1% 6 h post-admission. In our study, a ΔPPV cutoff below 7.6% is also significant for identifying patients at increased risk of early organ function deterioration and mortality. The lower threshold in our study may reflect differences in patient severity but collectively these findings reinforce ΔPPV as a clinically meaningful indicator of microcirculatory compromise and poor prognosis.

Microvascular endothelial cells are essential for regulating microcirculation function and maintaining vascular integrity, and they are significantly affected during sepsis. Recent clinical studies have frequently detected high concentrations of vascular endothelial shedding biomarkers in patients with sepsis-induced ARDS. Preliminary research by Kajita et al. (28) also highlights the close association between persistently elevated vascular endothelial shedding biomarkers levels and sepsis-induced ARDS. Our findings indicate more severe and prolonged microcirculation damage in patients with early-stage organ dysfunction, suggesting that prompt repair of the microcirculatory perfusion could enhance patient outcomes. The negative correlation between ΔPPV, LCR and ΔSOFA further emphasizes the relationship between microcirculatory disturbances and organ failure. In our research, the 28-day cumulative survival rate of patients without early organ function deterioration was significantly higher than that of patients with early organ function deterioration. This finding also underscores the importance for clinicians to monitor microvascular blood flow during resuscitation and to make active efforts to restore microvascular perfusion.

Our study has several limitations. First, this is a single-center, retrospective study with a relatively limited sample size. The retrospective design allows only for the identification of associations, not causal inference. Although bootstrap internal validation was used to assess model overfitting, the small sample size limits statistical power, particularly in multivariate logistic regression with multiple predictors, and increases the risk of spurious findings. Second, despite collecting baseline hemodynamics, laboratory data, and treatment details, we did not adjust for key therapeutic confounders (e.g., timing of antibiotics, source control, ventilator or vasoactive strategies), which may introduce residual confounding. Future large-scale, multicenter, prospective studies should collect data prospectively with different threshold predefined as a primary endpoint to validate our findings.

## 5 Conclusions

This study demonstrates that the change rate in the proportion of perfused vessels (ΔPPV) holds significant predictive value for early organ function deterioration in patients with septic shock and acute respiratory distress syndrome (ARDS). When combined with the lactate clearance rate (LCR), ΔPPV enhances predictive efficiency, establishing these metrics as effective indicators for evaluating disease progression and treatment outcomes. Monitoring sublingual microcirculation and its changes has emerged as a key focus in future clinical research, with the aim of enhancing treatment strategies for microcirculation.

## Data Availability

The raw data supporting the conclusions of this article will be made available by the authors, without undue reservation.
