# Supplementary material for: The value of proportion of perfused vessels change rate in the evaluation of early organ function deterioration in septic shock and ARDS
Source: Front Med (Lausanne). 2026 Jan 22;13:1733855. doi: 10.3389/fmed.2026.1733855 (PMC12872844; doi:10.3389/fmed.2026.1733855)
Supplement: Supplementary file 1 [file Table_1.docx]

**Table E1**

Distribution of ΔPPV in patients with mild, moderate and severe ARDS

| **Characteristics** |  | **Mild ARDS** | **Moderate ARDS** | **Severe ARDS** | ***P*** |
| --- | --- | --- | --- | --- | --- |
|  |  | **(*n*=26)** | **(*n*=34)** | **(*n*=7)** |  |
| PaO_2_/FiO_2_ | 0 h | 258.00（224.33,282.22） | 153.22(128.46,175.45) ^a^ | 97.00(91.11,98,14) ^a, b,^ | ＜0.001^*^ |
| HR (min-1) | 0 h | 98.50±7.50 | 99.00±8.30 | 100.29±9.38 | 0.873 |
|  | 6 h | 95.04±10.54 | 95.57±10.49 | 99.00±8.98 | 0.666 |
| CVP (cmH_2_O) | 0 h | 8.96±1.51 | 9.21±1.61 | 10.28±5.28 | 0.373 |
|  | 6 h | 8.96±1.95 | 9.65±1.84 | 9.14±1.68 | 0.363 |
| MAP (mmHg) | 0 h | 71.75±19.30 | 73.29±12.18 | 70.77±17.67 | 0.895 |
|  | 6 h | 79.78±10.71 | 80.60±12.60 | 81.20±22.00 | 0.956 |
| Lac (mmol/L) | 0 h | 6.92±3.78 | 6.53±3.15 | 7.94±3.63 | 0.609 |
|  | 6 h | 5.42±3.26 | 5.49±2.76 | 7.13±3.34 | 0.387 |
| PPV % | 0 h | 75.19±9.23 | 68.83±13.41 | 70.10±12.08 | 0.121 |
|  | 6 h | 80.76±9.93 | 76.83±13.93 | 71.64±11.54 | 0.184 |
| LCR | - | 19.51±18.43 | 16.96±14.44 | 10.50±8.40 | 0.403 |
| ΔPPV | - | 8.09(4.41,10.63) | 10.00(7.63,13.83) | 5.94(-0.88,9.06) ^b^ | **0.022^*^** |
| ΔSOFA≥1，n(%) |  | 15(57.69%) | 14(41.17%) | 5(71.43%) | 0.229 |

ΔSOFA , SOFA_24h-0h_ score ; a: compared with the mild group, b: compared with the moderate group，*P*＜0.05 **P*＜0.05.
