# Supplementary material for: The value of proportion of perfused vessels change rate in the evaluation of early organ function deterioration in septic shock and ARDS
Source: Front Med (Lausanne). 2026 Jan 22;13:1733855. doi: 10.3389/fmed.2026.1733855 (PMC12872844; doi:10.3389/fmed.2026.1733855)
Supplement: Supplementary file 2 [file Table_2.docx]

**Table E2**

Bootstrap-Validated Multivariate Regression Analysis (1000 Bootstraps)

|  | β | Bias | Std. Error | P | 95% CI (Lower) | 95% CI (Upper) |
| --- | --- | --- | --- | --- | --- | --- |
| LCR | -0.077 | -0.069 | 0.558 | 0.003 | -0.301 | -0.043 |
| PPV | -0.201 | -0.309 | 2.270 | 0.005 | -10.081 | -0.102 |
| PCT | 0.038 | 0.008 | 0.071 | 0.314 | -0.011 | 0.187 |
| Tbil | 0.024 | 0.019 | 0.169 | 0.085 | -0.004 | 0.096 |
| Constant | 2.067 | 3.381 | 25.617 | 0.182 | 0.541 | 11.587 |

**Table E3**

Multivariate Logistic Regression Analysis of Related Factors (With Sensitivity Analysis)

|  | β | Bias | Std. Error | P | OR | 95% CI (Lower) | 95% CI (Upper) |
| --- | --- | --- | --- | --- | --- | --- | --- |
| LCR | -0.093 | 0.036 | 6.601 | 0.01 | 0.911 | 0.849 | 0.978 |
| PPV | -0.19 | 0.064 | 8.815 | 0.003 | 0.827 | 0.729 | 0.937 |
| PCT | 0.033 | 0.02 | 2.766 | 0.096 | 1.034 | 0.994 | 1.075 |
| Tbil | 0.011 | 0.016 | 0.421 | 0.516 | 1.011 | 0.979 | 1.044 |
| SOFA | 0.474 | 0.177 | 7.22 | 0.007 | 1.607 | 1.137 | 2.272 |
| Constant | -3.443 | 2.195 | 2.46 | 0.117 | 0.032 | - | - |
